# RIP3 Associates with RIP1, TRIF, MAVS, and Also IRF3/7 in Host Innate Immune Signaling in Large Yellow Croaker *Larimichthys crocea*

**DOI:** 10.3390/antibiotics10101199

**Published:** 2021-10-01

**Authors:** Pengfei Zou, Kaiqing Li, Ying Li, Yingjia Shen, Ziping Zhang, Yilei Wang

**Affiliations:** 1 Key Laboratory of Healthy Mariculture for the East China Sea, Ministry of Agriculture and Rural Affairs, Fisheries College, Jimei University, Xiamen 361021, China; ylwang@jmu.edu.cn; 2 College of the Environment and Ecology, Xiamen University, Xiamen 361102, China; kaiqing.li@oebiotech.com (K.L.); shenyj@xmu.edu.cn (Y.S.); 3 Key Laboratory of Estuarine Ecological Security and Environmental Health, Tan Kah Kee College, Xiamen University, Zhangzhou 363105, China; 4 College of Animal Science, Fujian Agriculture and Forestry University, Fuzhou 350002, China; zhangziping@fafu.edu.cn; 5 State Key Laboratory of Large Yellow Croaker Breeding, Ningde Fufa Fisheries Company Limited, Ningde 352103, China

**Keywords:** RIP3, TRIF, MAVS, IRF3, IRF7, large yellow croaker

## Abstract

Receptor-interacting protein 3 (RIP3) has been demonstrated to be a key regulator not only in cell death pathways including apoptosis and necroptosis but also in inflammation and host immune responses. In this study, a *RIP3* ortholog named *Lc-RIP3* is identified in large yellow croaker (*Larimichthys crocea*). The open reading frame (ORF) of *Lc-RIP3* is 1524 bp long and encodes a protein of 507 amino acids (aa). The deduced *Lc*-RIP3 protein has an N-terminal kinase domain and a C-terminal RHIM domain, and the genome organization of *Lc-RIP3* is conserved in teleosts with 12 exons and 11 introns but is different from that in mammals, which comprises 10 exons and 9 introns. Confocal microscopy revealed that *Lc*-RIP3 is a cytosolic protein. The expression analysis at the mRNA level indicated that *Lc-RIP3* is ubiquitously distributed in various tissues/organs, and could be up-regulated under poly I:C, LPS, PGN, and *Pseudomonas plecoglossicida* stimulation in vivo. Notably, *Lc*-RIP3 could induce NF-κB but not IRF3 activation. In addition, *Lc*-RIP3 co-expression with *Lc*-TRIF, *Lc*-MAVS, or *Lc*-IRF3 significantly abolishes the activation of NF-κB but enhances the induction of IRF3 activity. Moreover, NF-κB activity could be up-regulated when *Lc*-RIP3 is co-expressed with *Lc*-RIP1 or *Lc*-IRF7. These results collectively indicate that *Lc*-RIP3 acts as an important regulator in host innate immune signaling in teleosts.

## 1. Introduction

Teleosts have evolved both innate and adaptive immune systems to provide protection against pathogen invasion and tissue damage through a variety of defense responses including the induction of interferons (IFNs), inflammatory cytokines, chemokines, and the activation of cell death pathways [1,2,3]. The innate immunity has been demonstrated as the first line in the host immune defense, which is initiated by the recognition of pathogen-associated molecular patterns (PAMPs) or damage-associated molecular patterns (DAMPs) through a series of receptors termed pattern recognition receptors (PRRs) including toll-like receptors (TLRs), nucleotide-binding oligomerization domain (NOD)-like receptors (NLRs), retinoic acid inducible gene I (RIG-I)-like receptors (RLRs), C-type lectin receptors (CLRs), and absent in melanoma 2 (AIM2)-like receptors (ALRs), which finally activate the host immune response through an interaction with the downstream signaling molecules [4,5].

Receptor-interacting protein 3 (RIP3), which belongs to the receptor-interacting protein (RIP) kinase family, contains an N-terminal serine/threonine kinase domain and a C-terminal RIP homotypic interaction motif (RHIM) domain, and is one of the key molecules that has been demonstrated to play important roles in not only cell death pathways but also inflammatory responses in the PRR-mediated signaling cascade [6,7,8]. As the third described RIP kinase family member, RIP3 was first identified as an apoptosis-inducing molecule [9,10]. Further studies have demonstrated that RIP3 also acts as a key regulator in necroptosis in which RIP3 could interact with RIP1 through their RHIM domains, leading to the phosphorylation of the downstream pseudokinase named mixed lineage kinase domain-like protein (MLKL) and then triggering necroptosis [6,7,8,11]. Additionally, studies also revealed that RIP3 was associated with RIP1 in the Toll/IL-1 receptor domain (TIR)-containing adaptor, inducing IFNβ (TRIF)-induced type I IFN signaling after the recognition of PAMPs by TLR3 or TLR4 [7,8,12]. RIP3 is also involved in the activation of the NOD-like receptor protein 3 (NLRP3)-dependent inflammasome complex and interleukin-1β (IL-1β) inflammatory responses [7,13].

Although the function of mammalian RIP3 in apoptosis, necroptosis, and inflammatory responses has been broadly and deeply characterized, studies of the roles of RIP3 in teleosts are still limited. To date, only a few orthologs of *RIP3* have been cloned and characterized such as in zebrafish (*Denio rerio*) [14], half-smooth tongue sole (*Cynoglossus semilaevis*) [15], and black carp (*Mylopharyngodon piceus*) [16]. It was revealed that teleost RIP3 exhibited highly conserved pro-apoptotic and pro-necroptotic functions in comparison with their counterparts in mammals [14,15]. Notably, a recent study of black carp showed that RIP3 could collaborate with RIP1 to inhibit mitochondrial antiviral signaling protein (MAVS)-mediated antiviral signaling [16], implying the multiple and complex functions of RIP3 in teleosts.

As an economically important marine aquaculture fish in eastern and southern China, the large yellow croaker (*Larimichthys crocea*) has been widely cultivated in southern China especially in the Fujian province. However, with the rapid development of the large yellow croaker farming industry, increasing outbreaks of infectious diseases caused by parasites (*Cryptocaryon irritans*, etc.), bacteria (*Pseudomonas plecoglossicida*, etc.), and viruses (iridovirus, etc.) have significantly influenced the industry and resulted in large economic losses [17,18,19]. Thus, it is important to understand the basic immune mechanisms against such invading pathogens, which is meaningful for further disease control and prevention. Notably, we have previously revealed TRIF- and MAVS-mediated immune signaling in large yellow croakers [20,21], and further investigations also showed that RIP1 was associated with TRIF and MAVS in the host innate immune signaling, suggesting the pivotal regulatory function of RIP1 in the host immune defense. To further delineate the function of the RIP family member in the innate immunity, a *RIP3* ortholog named *Lc-RIP3* was cloned and characterized in large yellow croakers in the present study. The conserved domains of *Lc*-RIP3, the multiple alignments of the protein sequence with other vertebrate RIP3 proteins, and the phylogenetic relationship were identified. The genome organization and subcellular localization as well as the expression patterns of *Lc-RIP3* in various organs/tissues and stimulation under PAMPs or a bacterial pathogen infection were also examined. Notably, the present study assayed the association of *Lc*-RIP3 with TRIF, MAVS, interferon regulatory factor 3 (IRF3), and IRF7 in NF-κB and IRF3 signaling activation, which throws new light on understanding the roles of RIP3 in the regulation of the host immune response in teleosts.

## 2. Results

### 2.1. Identification and Sequence Analysis of RIP3 in Large Yellow Croakers

**Figure 1:**
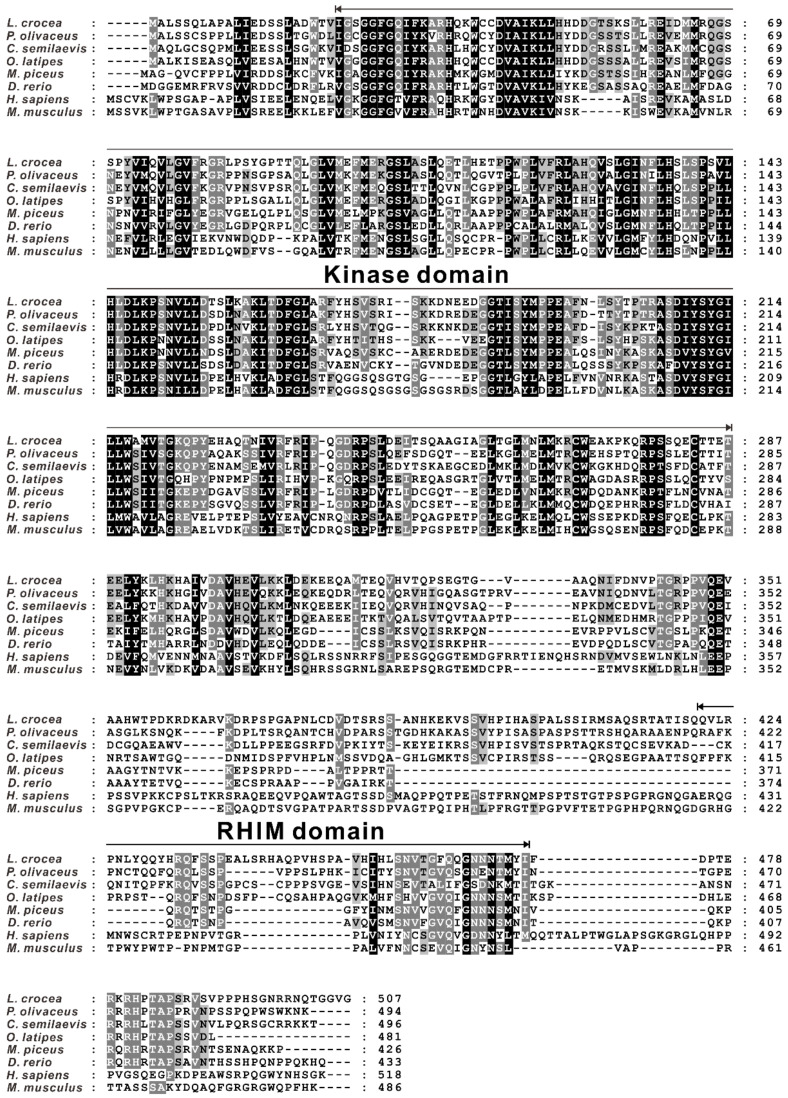
Multiple alignment of the protein sequence of *Lc*-RIP3 with other vertebrate RIP3. The amino acid sequence of *Lc*-RIP3 was compared with other vertebrate RIP3 by using Clustal X and the GeneDoc program. The black shaded areas indicate positions where all the sequences share the same amino acid residue whereas the gray and light gray shaded areas denote the conservative and semi-conservative amino acid substitutions, respectively. The kinase domain and RHIM domain are indicated with black arrows.

To explore the function of RIP3 in the immunity of large yellow croakers, the full-length open reading frame (ORF) of *RIP3* was cloned from the gill of a large yellow croaker and named as *Lc-RIP3* (GenBank accession No. MZ574078). The identified ORF of *Lc*-*RIP3* consisted of 1524 nucleotides, encoding a protein of 507 amino acids (aa). Based on the analysis of the conserved domain by using SMART, it was found that *Lc*-RIP3 contained an N-terminal kinase domain (23–287 aa) and an intermediate RIP homo interaction motif (RHIM, 421–473 aa) (Figure 1).

The amino acid sequence of *Lc*-RIP3 was subjected to multiple alignments with the RIP3 of other vertebrates including Japanese flounders (*Paralichthys olivaceus*), half-smooth tongue soles, medaka (*Oryzias latipes*), black carp, zebrafish, humans (*Homo sapiens*), and mice (*Mus musculus*), which revealed a relatively conservative N-terminal kinase domain in fish and mammals whereas the RHIM domain was less conserved (Figure 1). Additionally, the amino acid sequence of *Lc*-RIP3 had a similarity of 74% with the RIP3 of Japanese flounders, 67% with half-smooth tongue soles, 66% with medaka, 55% with black carp, and 52% with zebrafish, showing a relatively strong homology between different fish whereas compared with the RIP3 of mammals, the level of similarity decreased with a similarity of 40% to that in humans and 39% in mice (Table 1).

**Figure 2:**
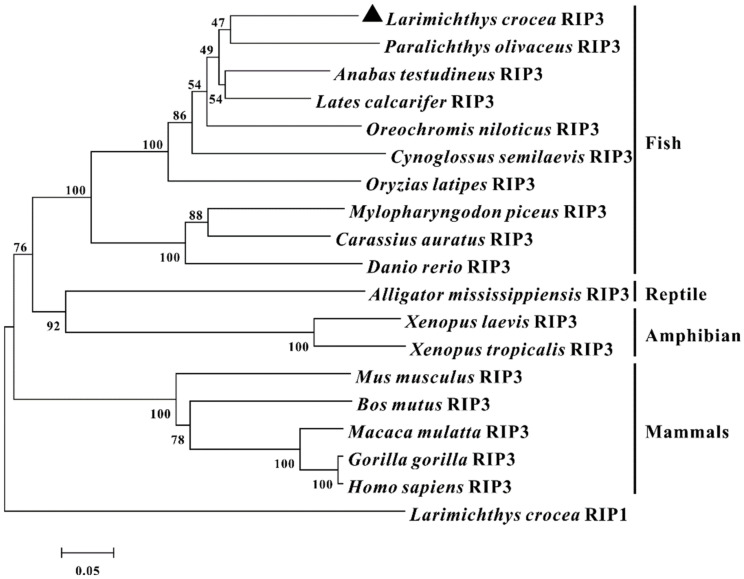
Phylogenetic analysis of vertebrate RIP3. The phylogenetic tree was constructed according to the alignment of amino acid sequences using the neighbor-joining method by MEGA 7.0 software with 10,000 replications in the bootstrap test. The GenBank accession numbers for the amino acid sequences used are as follows: *L. crocea* RIP3 (MZ574078), *P. olivaceus* RIP3 (XP_019967771.1), *A. testudineus* RIP3 (XP_026208769.1), *Lates calcarifer* RIP3 (XP_018543261.1), *C. semilaevis* RIP3 (XP_008316289.1), *Oreochromis niloticus* RIP3 (XP_003458818.2), *O. latipes* RIP3 (XP_023821938.1), *M. piceus* RIP3 (QXJ87326.1), *Carassius auratus* RIP3 (XP_026122240.1), *D. rerio* RIP3 (XP_001343827.1), *Alligator mississippiensis* RIP3 (XP_019343266.1), *Xenopus laevis* RIP3 (XP_018086823.1), *X. tropicalis* RIP3 (XP_002937800.1), *M. musculus* RIP3 (NP_064339.2), *Bos mutus* RIP3 (XP_014338619.1), *Macaca mulatta* RIP3 (XP_001114079.1), *Gorilla gorilla* RIP3 (XP_004055062.1), *H. sapiens* RIP3 (NP_006862.2), and *L. crocea* RIP1 (MZ274348).

To investigate the phylogenic relationship of RIP3 in vertebrates, a phylogenetic tree was constructed using the neighbor-joining method in Molecular Evolutionary Genetics Analysis (MEGA) version 7.0 software based on the amino acid sequences of 19 species of vertebrates. The results showed that these RIP3 orthologs and the large yellow croaker RIP1 were divided into two groups with different mammalian RIP3 gathering in one clade with a 100% bootstrap support level and teleost, reptile, and amphibian RIP3 gathering in another clade in which *Lc*-RIP3 clustered together with the Japanese flounder RIP3 (Figure 2).

### 2.2. Genomic Organization of RIP3 Genes in Vertebrates

**Figure 3:**
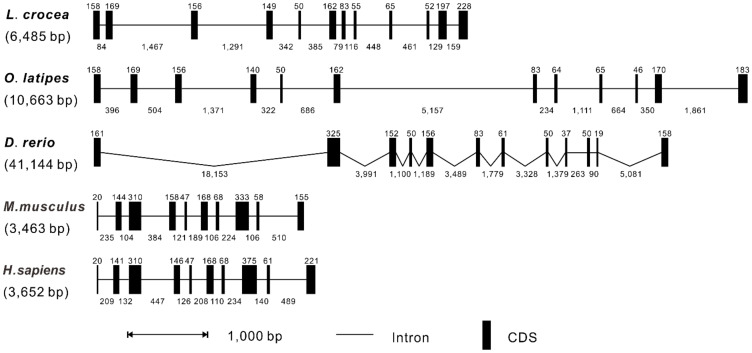
Genomic organization comparison of *Lc*-*RIP3* with other vertebrates. The genomic organization of the *Lc*-*RIP3* gene from various vertebrate species was compared and analyzed including *O. latipes*, *D. rerio*, *M. musculus*, and *H. sapiens*. Black boxes indicate the coding sequences (CDS) with the length of exons in base pairs (bp) shown above the black boxes. The lines denote the introns with the lengths of the introns in bp shown below the lines. The genomic DNA sequence locations and their accession numbers were obtained from the GenBank database: *L. crocea*, NC_040024.1 (8549234–8555718); *O. latipes*, NC_019876.2 (21473539–21484201); *D. rerio*, NC_007118.7 (1324910–1366053); *M. musculus*, NC_000080.7 (56022653–56026093); and *H. sapiens*, NC_000014.9 (24336175–24339826).

According to the genomic sequences of *L. crocea*, *O. latipes*, *D. rerio*, *M. musculus*, and *H. sapiens* in the NCBI database, the *RIP3* genomic structures of vertebrates were compared by Splign software. It was revealed that the genomic sequences of *RIP3* had a range of 3463 bp to 41,144 bp in length from teleosts to mammals with the shortest identified in mice and the longest in zebrafish, respectively (Figure 3). The alignment of the cDNA sequence with the corresponding genomic sequence showed that the exon-intron organization of *RIP3* in *L. crocea* comprised 12 exons and 11 introns, which was similar to that found in *O. latipes* and *D. rerio*, whereas *M. musculus* and *H. sapiens* comprised 10 exons and 9 introns.

### 2.3. Subcellular Localization of Lc-RIP3

**Figure 4:**
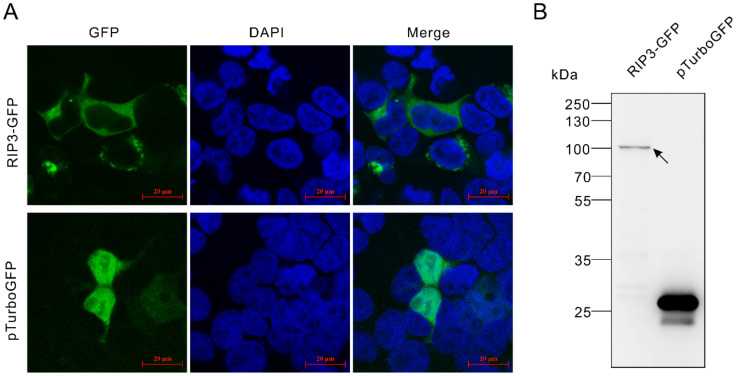
Subcellular localization of *Lc*-RIP3. (**A**) HEK 293T cells were transfected with pTurbo-RIP3-GFP and pTurboGFP-N (vector control), respectively. At 24 h post-transfection, the cells were stained with DAPI and then examined and photographed under a confocal microscope. (**B**) The confirmation of the successful expression of the RIP3-GFP and pTurboGFP fusion proteins was conducted by a Western blotting analysis using an Anti-TurboGFP antibody.

The full-length ORF of *Lc*-*RIP3* was cloned and constructed into the pTurboGFP-N green fluorescent expression vector and the subcellular localization of pTurbo-RIP3-GFP was detected by confocal microscopy. The results revealed that the pTurbo-RIP3-GFP fusion protein was distributed in the cytoplasm with apparent brilliant green spots identified around the nucleus (Figure 4A). In contrast, pTurboGFP was present in the whole cell including the nucleus (Figure 4A). The expressions of pTurbo-RIP3-GFP and the pTurboGFP fusion proteins were verified by a Western blotting analysis using an Anti-TurboGFP antibody, which confirmed the successful expression of the pTurbo-RIP3-GFP fusion protein (Figure 4B).

### 2.4. Expression Pattern Analysis of Lc-RIP3 in Organs/Tissues

**Figure 5:**
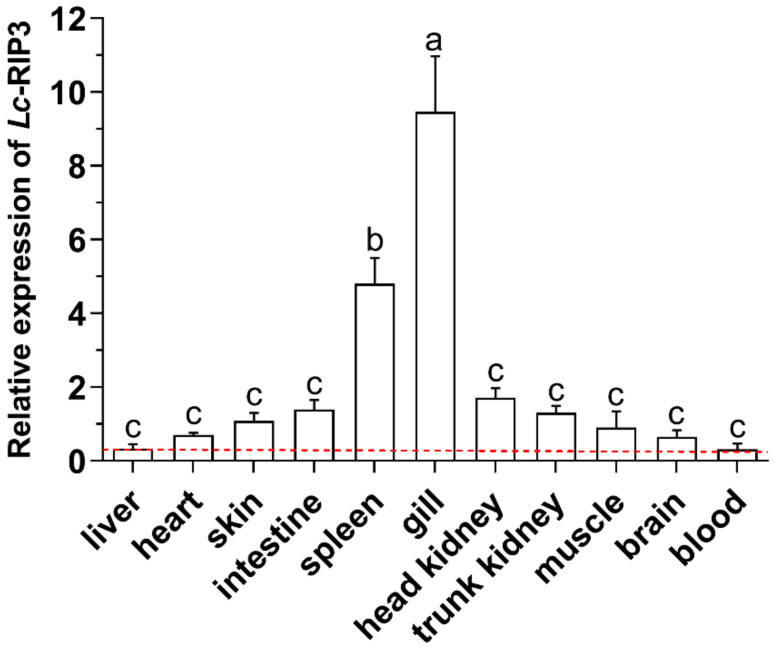
Distribution pattern of *Lc*-*RIP3* mRNA in large yellow croakers. The mRNA expression levels of *Lc*-*RIP3* in various organs/tissues of healthy fish (*n* = 6) were detected by qRT-PCR with the results normalizing the expression of *β*-actin. The expression levels in 11 organs/tissues were set as multiples relative to the average expression of *Lc*-*RIP3* in all tissues and the baseline of the lowest expression level was marked with a red dotted line. All data are shown as mean ± SE (*n* = 6). A statistical analysis was performed using a one-way ANOVA followed by a Duncan’s multiple range test. Different superscripts indicate statistically different results (*p* < 0.05) and the same superscript indicates no statistical differences between groups.

The expression patterns of *Lc-RIP3* mRNA in various organs/tissues of large yellow croakers including the gill, spleen, head kidney, intestine, trunk kidney, skin, muscle, heart, brain, liver, and blood were investigated by using quantitative real-time PCR (qRT-PCR). The results showed that the *Lc-RIP3* transcripts were ubiquitously expressed in all organs/tissues with the highest and lowest expression levels detected in the gill and blood of healthy fish, respectively (Figure 5).

**Figure 6:**
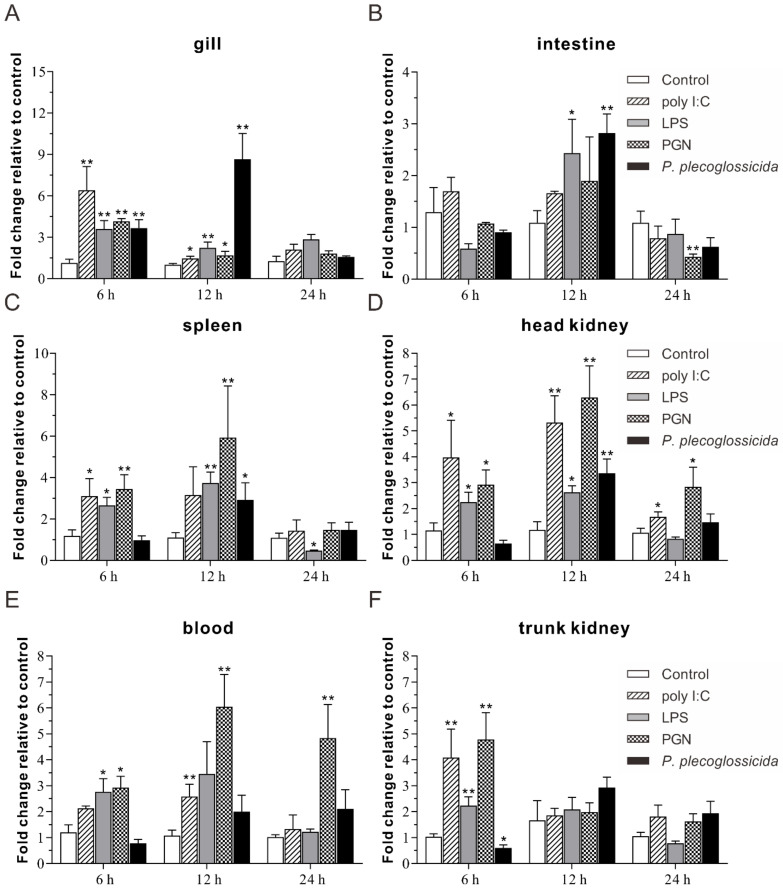
Expression patterns of *Lc-RIP3* in response to stimulations of poly I:C, LPS, PGN, and *P. plecoglossicida*. A healthy large yellow croaker (five groups) was injected intraperitoneally with 100 μL of PBS (control), poly I:C (1 mg/mL), LPS (0.5 mg/mL), PGN (1 mg/mL), or a *P. plecoglossicida* suspension in PBS (5 × 10^5^ CFU/mL), respectively. The mRNA expression levels of *Lc-RIP3* in the gill (**A**), intestine (**B**), spleen (**C**), head kidney (**D**), blood (**E**), and trunk kidney (**F**) were examined by qRT-PCR at indicated time points post-stimulation with the results calculated by the normalization to the expression of *β*-actin and then recorded as a fold change compared with the control group at the same time point. All of the data are shown as mean ± SE (*n* = 6) with bars representing the SE. * *p* < 0.05, ** *p* < 0.01.

To further understand the mRNA expression profiles of *Lc-RIP3* in the host innate immune response, the changes in the expression levels of *Lc-RIP3* in different tissues including the gill, intestine, spleen, head kidney, blood, and trunk kidney under polyinosinic-polycytidylic acid potassium salt (poly I:C), peptidoglycan (PGN), lipopolysaccharides (LPS), or *P. plecoglossicida* stimulation were detected by qRT-PCR. The results showed that the expression levels of *Lc-RIP3* in the gill, intestine, spleen, and head kidney were significantly increased in response to a *P. plecoglossicida* infection with a 3.7- and 8.7-fold increase at 6 and 12 hpi in the gill (Figure 6A), respectively, and a 2.8-, 2.9-, and 3.4-fold increase at 12 hpi in the intestine, spleen, and head kidney (Figure 6B–D), respectively. Under a poly I:C stimulation, the expression levels of *Lc-RIP3* in the gill, spleen, head kidney, blood, and trunk kidney were significantly up-regulated with 6.4- and 1.5-fold increase at 6 and 12 hpi in the gill (Figure 6A), respectively, a 3.1- and 4.1-fold increase at 6 hpi in the spleen and trunk kidney (Figure 6C,F), respectively, a 4.0-, 5.3-, and 1.7-fold increase at 6, 12, and 24 hpi in the head kidney (Figure 6D), respectively, and a 2.6-fold increase at 12 hpi in the blood (Figure 6E). The expression level of *Lc-RIP3* also significantly increased under LPS stimulation, which showed a 3.6- and 2.2-fold, a 2.7- and 3.7-fold, and a 2.2- and 2.6-fold increase at 6 and 12 hpi in the gill, spleen, and head kidney (Figure 6A,C,D), respectively, a 2.4-fold increase at 12 hpi in the intestine (Figure 6B), and a 2.8- and 2.2-fold increase at 6 hpi in the blood and trunk kidney (Figure 6E,F), respectively. In addition, the expression level of *Lc-RIP3* was significantly up-regulated under PGN stimulation with a 4.2- and 1.7-fold and 3.4- and 5.9-fold increase at 6 and 12 hpi in the gill and spleen (Figure 6A,C), respectively, a 2.9-, 6.3-, and 2.8-fold and a 2.9-, 6.0-, and 4.8-fold increase at 6, 12, and 24 hpi in the head kidney and blood (Figure 6D,E), respectively, and a 4.8-fold increase at 6 hpi in the trunk kidney (Figure 6F). Nevertheless, the expression level of *Lc-RIP3* was significantly down-regulated at 24 hpi in the intestine and spleen under PGN and LPS stimulation and at 6 hpi in the trunk kidney under the *P. plecoglossicida* infection, respectively (Figure 6B,C,F).

### 2.5. The Role of Lc-RIP3 in the Regulation of NF-κB Signaling

**Figure 7:**
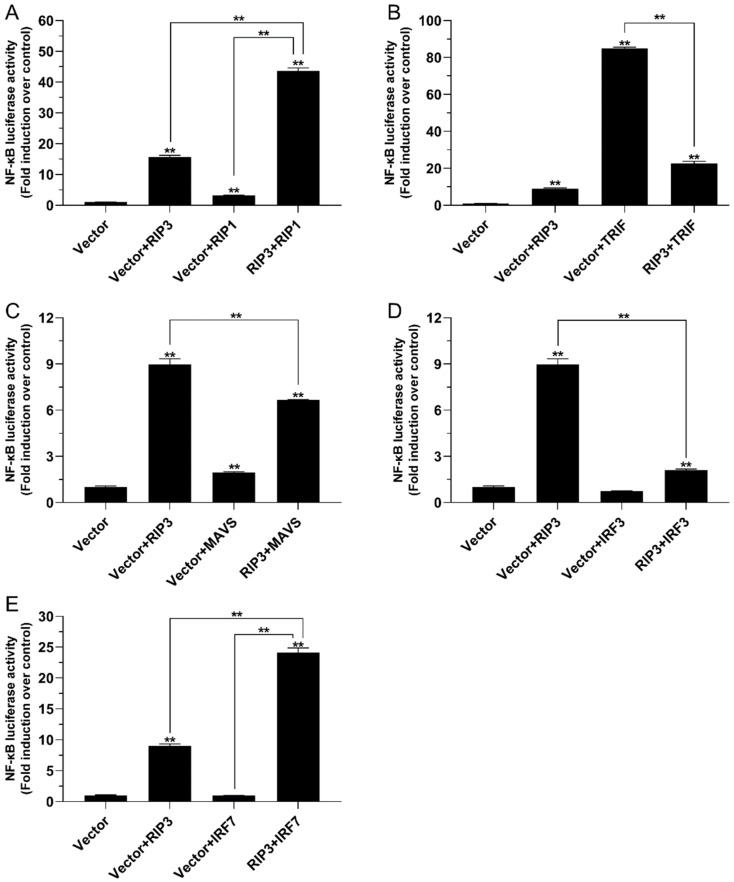
The role of *Lc*-RIP3 in the regulation of NF-κB signaling. The associations of *Lc*-RIP3 with *Lc*-RIP1 (**A**), *Lc*-TRIF (**B**), *Lc*-MAVS (**C**), *Lc*-IRF3 (**D**), and *Lc*-IRF7 (**E**) in NF-κB promoter activation were analyzed by luciferase reporter assays. HEK 293T cells were co-transfected with 100 ng of pNF-κB-luc and 10 ng of pRL-TK together with 100 ng of pcDNA3.1-RIP3, pcDNA3.1-RIP1, pcDNA3.1-TRIF, pcDNA3.1-MAVS, pcDNA3.1-IRF3, and pcDNA3.1-IRF7 alone or in a combination of two with the total amount of transfected plasmids balanced by the pcDNA3.1 empty vector for each transfection. The cells were then harvested and lysed for the measurement of luciferase activities at 24 h post-transfection. All data were presented as mean ± SE from three independent experiments with the bars representing the SE. ** *p* < 0.01.

To describe the regulation function of *Lc*-RIP3 in NF-κB signaling, the expression plasmids of *Lc*-RIP3 and those of *Lc*-RIP1, *Lc*-TRIF, *Lc*-MAVS, *Lc*-IRF3, and *Lc*-IRF7 together with NF-κB reporter plasmids were co-transfected into HEK 293T cells and then detected by dual-luciferase reporter assays. The results showed that the overexpression of *Lc*-RIP3 or *Lc*-RIP1 alone could significantly provoke NF-κB activation with *Lc*-RIP3 inducing a relatively higher level of NF-κB promoter activity. The co-expression of *Lc*-RIP3 with *Lc*-RIP1 induced a remarkably higher level of NF-κB promoter activity compared with the overexpression of *Lc*-RIP3 or *Lc*-RIP1 alone, which had a 44-fold increase compared with the control group (Figure 7A). Moreover, the co-expression of *Lc*-RIP3 with *Lc*-IRF7 also significantly increased the activation of the NF-κB promoter compared with the overexpression of *Lc*-RIP3 alone although the *Lc*-IRF7 overexpression showed no significant effect in the induction of the NF-κB promoter activity (Figure 7E). However, the co-expression of *Lc*-RIP3 with *Lc*-TRIF significantly reduced the NF-κB promoter activity compared with the overexpression of *Lc*-TRIF alone (Figure 7B). In addition, the co-expression of *Lc*-RIP3 with *Lc*-MAVS or *Lc*-IRF3 significantly down-regulated the level of NF-κB activation in comparison with the only transfection of *Lc*-RIP3 alone. The overexpression of *Lc*-IRF3 exhibited no significant effect in provoking NF-κB activation (Figure 7C,D).

### 2.6. The Role of Lc-RIP3 in the Regulation of IRF3 Signaling

**Figure 8:**
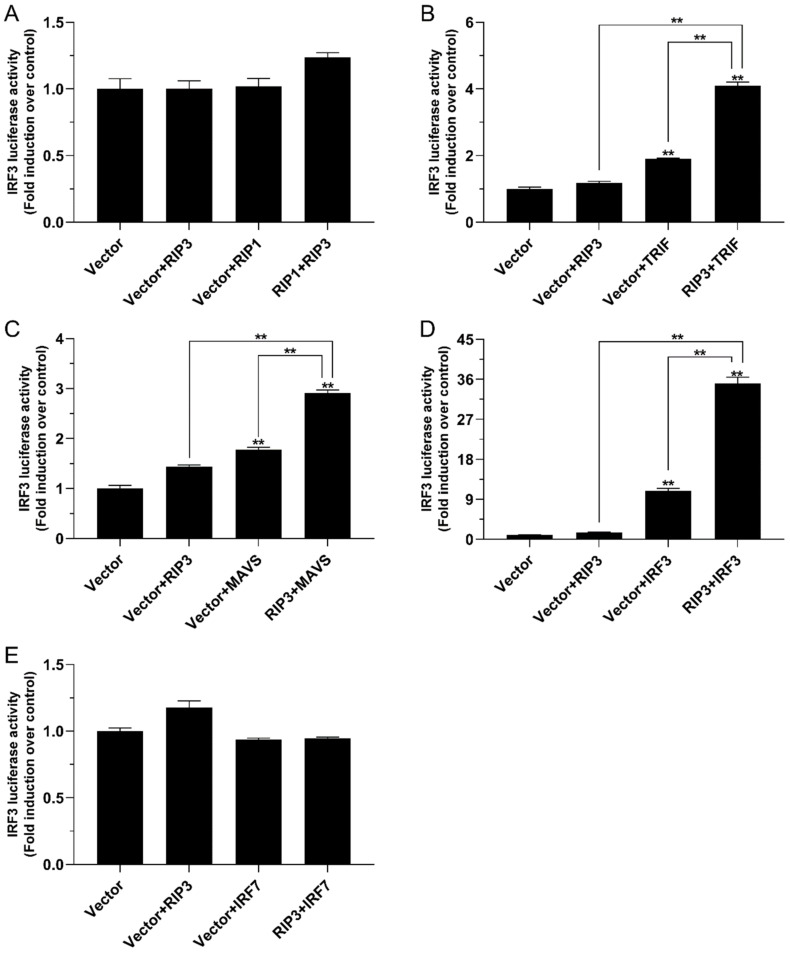
The role of *Lc*-RIP3 in the regulation of IRF3 signaling. The associations of *Lc*-RIP3 with *Lc*-RIP1 (**A**), *Lc*-TRIF (**B**), *Lc*-MAVS (**C**), *Lc*-IRF3 (**D**), and *Lc*-IRF7 (**E**) in IRF3 promoter activation were analyzed by luciferase reporter assays. HEK 293T cells were co-transfected with 100 ng of pGL4-IRF3-pro and 10 ng of pRL-TK together with 100 ng of pcDNA3.1-RIP3, pcDNA3.1-RIP1, pcDNA3.1-TRIF, pcDNA3.1-MAVS, pcDNA3.1-IRF3, and pcDNA3.1-IRF7 alone or in a combination of two as described above. The luciferase activities were then measured from the cell lysates at 24 h post-transfection. All data were expressed as mean ± SE from three independent experiments with bars representing the SE. ** *p* < 0.01.

To investigate the function of *Lc*-RIP3 in the IRF3-mediated signaling pathway, HEK 293T cells were co-transfected with the expression plasmids described above together with IRF3 reporter plasmids and used for dual-luciferase reporter assays. The results revealed that although the overexpression of *Lc*-RIP3 alone had no significant effect on IRF3 promoter activation, the co-expression of *Lc*-RIP3 with *Lc*-TRIF or *Lc*-MAVS significantly increased the activation of the IRF3 promoter activity induced by *Lc*-TRIF or *Lc*-MAVS alone (Figure 8B,C). Notably, it was revealed that the single overexpression of *Lc*-IRF3 significantly induced the activation of the IRF3 promoter activity and the co-expression of *Lc*-RIP3 with *Lc*-IRF3 induced a remarkably higher level of the IRF3 promoter activity compared with the overexpression of *Lc*-IRF3 alone, which was a 35-fold increase compared with the control group (Figure 8D). However, the co-expression of *Lc*-RIP3 with *Lc*-RIP1 or *Lc*-IRF7 had no significant effect on the activation of the IRF3 promoter activity (Figure 8A,E).

## 3. Discussion

First reported in 1999 in humans, RIP3 was initially identified as an apoptosis-inducing kinase [9,10]. Subsequent investigations revealed the key roles of RIP3 in necroptosis and also in the activation of inflammatory responses [6,7,8]. Additionally, accumulating evidence has shown that RIP3 has an important function in host antiviral and also antibacterial responses [22,23]. However, few studies have focused on how RIP3 functions in the host innate immune signaling. Herein, the ortholog of RIP3 was cloned and identified in large yellow croakers; the regulatory function of RIP3 with RIP1, TRIF, MAVS, IRF3, and IRF7 in inducing NF-κB and IRF3 activation was also investigated. The results of the present study have provided novel findings of RIP3 that are involved in the host innate immune signaling pathway in teleosts.

An expression pattern analysis showed that *Lc-RIP3* was constitutively expressed in all the examined organs/tissues in healthy large yellow croakers with the highest expression in the gill, which was consistent with our data that *Lc-RIP1* was highly expressed in the gill (unpublished results). In addition, many immune-related genes such as *TRIF*, *TRAF3*, *IL-17*, and *TLR21* were also found to exhibit the highest expression level in the gill of large yellow croakers [20,24,25,26], implying the important function of the gill-associated lymphoid tissue (GIALT) in the mucosa immunity of teleosts [27]. In addition, the expression patterns of *Lc-RIP3* in the gill, intestine, spleen, head kidney, blood, and trunk kidney were significantly up-regulated in response to poly I:C, LPS, PGN, and *P. plecoglossicida* stimulation, which were consistent with studies of other teleosts that RIP3 in half-smooth tongue sole could be induced under *Vibrio anguillarum* or poly I:C treatment [15] and black carp *RIP3* could also be activated in response to poly I:C or LPS stimulation [16], suggesting the possible roles of RIP3 in both bacterial and viral-induced immune responses in teleosts.

In mammals, RIP3 has been demonstrated to interact with RIP1 through the RHIM domains to form a protein complex to activate downstream signaling, which leads to the activation of necroptosis [6,28]. It has also been revealed in teleosts that black carp RIP3 could interact with RIP1 [16]. Additionally, the results of the present study showed that *Lc*-RIP3 as well as *Lc*-RIP1 overexpression could significantly induce NF-κB activation, consistent with the reports that mammalian RIP1 and RIP3 could activate NF-κB [10,29,30,31]. The co-expression of *Lc*-RIP3 with *Lc*-RIP1 could significantly enhance NF-κB activation, implying the possible association of *Lc*-RIP3 with *Lc*-RIP1 in NF-κB-mediated signaling. In addition to the interaction with RIP1, RIP3 has been revealed to interact with other RHIM-containing proteins such as TRIF [6,32]. TRIF was first identified as an adaptor downstream of TLR3 and TLR4 that initiates the activation of NF-κB and the IRF3-mediated production of type I IFNs through the recognition of PAMPs [33,34]. Further investigations in teleosts indicated that TRIF can act as an important adaptor in TLR3-, TLR19-, and TLR22-mediated signaling pathways [35,36]. Our previous results also showed that a large yellow croaker TRIF overexpression is sufficient for the induction of NF-κB, IRF3, IRF7, and also type I IFN promoter activation [20]. Notably, the results of the present study showed that the co-expression of *Lc*-RIP3 with *Lc*-TRIF could significantly abolish the induction of NF-κB activity but enhance IRF3 activation although the overexpression of *Lc*-RIP3 alone exhibited no effect in IRF3 activation, indicating the regulation of *Lc*-RIP3 in *Lc*-TRIF-mediated signaling. Additionally, as an important adaptor, MAVS functions dominantly in RLRs-mediated antiviral signaling [21,37]. Interestingly, our results indicated that the co-expression of *Lc*-RIP3 with *Lc*-MAVS significantly down-regulated the induction of the NF-κB activity but up-regulated the IRF3 activation. Similarly, studies in other teleosts also revealed the association of RIP3 with MAVS in the host immune response although the results showed a little difference in which black carp RIP3 collaborated with RIP1 to inhibit MAVS-mediated signaling [16].

As key transcription factors, IRF3 and IRF7 function in TRIF, MAVS, and the stimulator of IFN genes (STING)-related signaling cascade and trigger the production of type I IFNs [38,39]. Thus, it is of great importance to understand the association of *Lc*-RIP3 with IRF3/7 in type I IFN signaling. Notably, our results in large yellow croakers found that the co-expression of *Lc*-RIP3 with *Lc*-IRF3 could significantly suppress the activation of NF-κB but enhance the IRF3 activation whereas the co-expression of *Lc*-RIP3 with *Lc*-IRF7 significantly enhanced the activation of NF-κB. These results collectively indicate the diverse roles of *Lc*-RIP3 in IRF3- and IRF7-mediated NF-κB and IRF3 activation. So far, little evidence has been proposed on the regulation of RIP3 in IRF3/7-mediated signaling; it is therefore suggested that further studies should focus on the mechanism that *Lc*-RIP3 is involved in the regulation of the IRF3/7-mediated signaling pathway.

## 4. Materials and Methods

### 4.1. Fish, Cell Lines, and Transfection

Large yellow croakers (weight 60 ± 15 g; length 18 ± 1.5 cm) were obtained from Ningde Fufa Fishing Co., Ltd., Ningde, Fujian Province, China. They were maintained in laboratory recirculating seawater systems. After acclimating for two weeks, the healthy fish were used for the subsequent experiments as per our previous reports [20,24].

Human embryonic kidney 293T (HEK 293T) cells were maintained in Dulbecco’s Modified Eagle Medium (DMEM) supplemented with 10% fetal bovine serum (FBS, Invitrogen-Gibco) and 100 U/mL penicillin and streptomycin at 37 °C in a 5% CO_2_ incubator as described previously [40]. The transfection of plasmids into the HEK 293T cells was performed by using Lipofectamine 3000 (Invitrogen, Carlsbad, CA, USA) according to the standard protocols as indicated in the manuals of the manufacturers.

### 4.2. Gene Cloning and Construction of Plasmids

Based on the transcriptome data of large yellow croaker *RIP3* in the NCBI database (GenBank accession No. XM_027288056.1), the full-length ORF of large yellow croaker *RIP3* was amplified by using specific primers and Ex-Taq (Takara, Dalian, China) with cDNA synthesized from the gill used as a template and then inserted into pcDNA3.1/*myc*-His (−) A vector (Invitrogen, Carlsbad, CA, USA) for the overexpression studies. For the subcellular localization analysis, the full-length ORF of *RIP3* was also cloned into the pTurboGFP-N vector (Evrogen, Moscow, Russia). In addition, the entire ORFs of large yellow croaker *RIP1*, *TRIF*, *MAVS*, *IRF3*, and *IRF7* were also cloned and constructed into pcDNA3.1/*myc*-His (−) A vector, respectively. All plasmid constructs were confirmed by a sequencing analysis in Sangon Biotech Co., Ltd. (Shanghai, China). The primers with the restricted enzyme cutting sites are listed in Table 2.

### 4.3. Immune Stimulation and qRT-PCR

For the tissue distribution pattern analysis of *RIP3* in large yellow croakers, various organs/tissues (gill, spleen, head kidney, intestine, trunk kidney, skin, muscle, heart, brain, liver, and blood) were dissected from six healthy fish, which were anesthetized in 0.01% eugenol and then put into liquid nitrogen immediately for RNA extraction.

To examine the expression patterns of *RIP3* under various immune stimulations, five groups of healthy fish were injected with different PAMPs and bacteria as described previously [20,24]. The control group of fish was injected intraperitoneally with 100 μL PBS. Simultaneously, the challenge groups of fish were injected with 100 μL poly I:C (a double-stranded homopolymer that simulates viral dsRNA) (P9582, Sigma, 1 mg/mL), 100 μL PGN (a major cell wall component of Gram-positive bacteria) (69554, Sigma, from *Bacillus subtilis*, 1 mg/mL), 100 μL LPS (a major biologically active agent of Gram-negative bacterial) (L3024, Sigma, from *Escherichia coli* O111:B4, 0.5 mg/mL), or a 100 μL suspension of *P. plecoglossicida* (a bacterial pathogen causing visceral granulomas in farmed large yellow croakers) (5 × 10^5^ CFU/mL) in PBS, respectively. At 6, 12, and 24 h post-injection (hpi), various organs/tissues including the gill, intestine, spleen, head kidney, blood, and trunk kidney were collected for total RNA extraction from six fish of each group.

The total RNA was isolated with an Eastep^TM^ Super Total RNA Extraction Kit (Promega, Beijing, China) followed by the determination of the quality by electrophoresis and an OD260/280 ratio, and then reverse transcribed into cDNA by using a first stand cDNA synthesis kit (RevertAid First Stand cDNA Synthesis Kit, #K1622, Thermo Scientific™) according to the manufacturer’s instructions. The synthetic cDNA was then kept at −20 °C and used as a template for ORF cloning of the target genes or the qRT-PCR analysis.

The qRT-PCR was performed on a Roche LightCycler^®^ 480 II quantitative real-time detection system (Roche, Switzerland) by using Go Taq^®^ qPCR Master Mix (Promega, Madison, WI, USA). The cycling conditions were as follows: 5 min for the initial denaturation at 95 °C followed by 40 cycles of 20 s at 95 °C, 15 s at 58 °C, and 15 s at 72 °C. All reactions were performed in triplicate in a 384-well plate. The relative expression levels of *Lc-RIP3* were normalized to *β*-actin and calculated using the 2^−ΔΔCt^ comparative Ct method [41]. The statistical analysis was conducted using a one-way analysis of variance (ANOVA) followed by a Duncan’s multiple range test with SPSS version 20. The expression data were represented as mean ± standard error (SE). An asterisk (*) on the pillar indicated the significant difference between the experimental and control groups (* *p* < 0.05; ** *p* < 0.01). All primers used for qRT-PCR are listed in Table 2.

### 4.4. Bioinformatics Analysis

The protein sequence prediction was performed using software at the ExPASy Bioinformatics Resource Portal (http://www.expasy.org/proteomics; accessed on 3 February 2021) and the conserved domain structures were predicted by using the Simple Modular Architecture Research Tool (SMART) (http://smart.embl.de; accessed on 24 February 2021) [42]. The sequences of the RIP3 of vertebrates were searched for by using the Basic Local Alignment Search Tool (BLAST) analysis tool of the National Center for Biotechnology Information website (NCBI, http://www.ncbi.nlm.nih.gov/blast; accessed on 14 January 2021). The sequence alignments were carried out using the Clustal X program followed by editing with GeneDoc software. The phylogenetic tree was constructed using the neighbor-joining method in MEGA version 7.0 [43].

### 4.5. Confocal Microscopy

To understand the subcellular localization of large yellow croaker RIP3, HEK 293T cells were seeded on sterilized coverslips in 6-well plates at a density of 2 × 10^5^ cells per well. The following day, the cells were transfected with 5 μg of plasmid constructs of pTurbo-RIP3-GFP or pTurboGFP-N (vector control) using Lipofectamine 3000 according to the manufacturer’s instructions. At 24 h post-transfection, the cells were washed with 1× PBS, fixed using 4% paraformaldehyde, and permeabilized with Triton X-100 (0.2% in PBS) for 15 min at room temperature, respectively. The coverslips were then washed with PBS and overlaid with one drop of the mounting medium (VECTASHIELDR Hard Set™ Mounting Medium with DAPI, Vector Laboratories, CA, USA). After staining with DAPI, the cells were visualized and imaged using a confocal microscope (Leica TCS SP8, Germany). The cells were subsequently collected and lysed in a RIPA buffer (Beyotime, Shanghai, China) containing protease inhibitors (Beyotime, Shanghai, China) for the detection of RIP3-GFP and the pTurboGFP fusion proteins using a Western blotting analysis as per our previous reports [20,24] with the primary antibody (Anti-TurboGFP antibody, Evrogen, CAT. #AB513) diluted at 1:5000.

### 4.6. Luciferase Activity Assay

To evaluate the association between large yellow croaker RIP3 and RIP1, TRIF, MAVS, IRF3, or IRF7 proteins, HEK 293T cells were seeded in 24-well plates at 1 × 10^5^ cells per well. The following day, the cells were transiently co-transfected with 100 ng of pNF-κB-luc (Clontech, Palo Alto, CA, USA) or pGL4-IRF3-pro (ZL201710457836.8), and 10 ng of pRL-TK (Promega, Madison, WI) together with the expression plasmids described above by using Lipofectamine 3000 with the total amount of transfected plasmids balanced by the pcDNA3.1 empty vector. At 24 h post-transfection, the cells were collected and lysed using a Dual-Luciferase Reporter System (Promega) according to the manufacturer’s instructions. The centrifuged supernatant was collected and used to measure the luciferase activities by a Promega GloMax^®^ 20/20 luminometer (Promega) with the firefly luciferase activity normalized to the *Renilla* luciferase activity. The results were expressed as a fold of the normalized luciferase activity in the cells transfected with various expression plasmids relative to the control cells transfected with the empty vector [20,24]. All values were expressed as the mean of three independent experiments and a statistical analysis was conducted using an ANOVA followed by a Duncan’s multiple range test by using SPSS version 20 with a probability level of *p* < 0.05 (*) or *p* < 0.01 (**) considered as statistically significant.

## 5. Conclusions

In conclusion, the present study cloned and functionally characterized RIP3 in large yellow croakers. *Lc*-RIP3 contained both a kinase domain and a RHIM domain, which was to an extent conserved in vertebrates. *Lc-RIP3* was broadly distributed in different organs/tissues, which could be up-regulated in response to poly I:C, LPS, PGN, and *P. plecoglossicida* stimulation. As a cytosolic protein, *Lc*-RIP3 overexpression could induce NF-κB but not IRF3 activation. Intriguingly, *Lc*-RIP3 overexpression could enhance *Lc*-RIP1 and *Lc*-IRF7-mediated NF-κB activation, and also *Lc*-TRIF and *Lc*-MAVS and *Lc*-IRF3-mediated IRF3 activation whereas it suppressed *Lc*-TRIF, *Lc*-MAVS, and *Lc*-IRF3-mediated NF-κB activation. These results collectively indicate the complicated regulation function of *Lc*-RIP3 in host innate immune signaling. Nevertheless, further studies are still needed to reveal the exact mechanism of teleost RIP3 in the regulation of the host immune response, which is important in understanding the critical roles of teleost RIP3 in host inflammation and immunity.

## Figures and Tables

**Table 1:** Amino acid identity and similarity among *Lc*-RIP3 and RIP3 for other vertebrates.

Common Name	Scientific Name	Accession No.	Length (aa)	Identity	Similarity
Japanese flounder	*Paralichthys olivaceus*	XP_019967771.1	494	61%	74%
Half-smooth tongue sole	*Cynoglossus semilaevis*	XP_008316289.1	496	52%	67%
Medaka	*Oryzias latipes*	XP_023821938.1	481	50%	66%
Black carp	*Mylopharyngodon piceus*	QXJ87326.1	426	37%	55%
Zebrafish	*Danio rerio*	XP_001343827.1	433	36%	52%
Human	*Homo sapiens*	NP_006862.2	518	23%	40%
Mouse	*Mus musculus*	NP_064339.2	486	24%	39%

**Table 2:** Primer sequences used in the present study.

Primer Name	Sequence (5′–3′)	Application
*Lc*-RIP3-F	ATGGCACTGTCCAGCCAGCT	*Lc*-RIP3 ORF cloning
*Lc*-RIP3-R	GCCAACTCCTCCTGTTTGGTTC	
pcDNA3.1-RIP3-F	CGGGATCCATGGCACTGTCCAGCCAGCT	pcDNA3.1-RIP3
pcDNA3.1-RIP3-R	GGGGTACCGCCAACTCCTCCTGTTTGGTTC	
pcDNA3.1-TRIF-F	CCGCTCGAGCGATGGCTAGCCGCGAGGGAGAAGA	pcDNA3.1-TRIF
pcDNA3.1-TRIF-R	CGGGGTACCTTGCTCATCTAAATCATCT	
pcDNA3.1-MAVS-F	CGCGGATCCATGGCTTCGTTTGCCAGAGACAG	pcDNA3.1-MAVS
pcDNA3.1-MAVS-R	CCCAAGCTTGTTCTTAAACTTCCACG	
pcDNA3.1-IRF3-F	CCGGAATTCCAATGGCTTCTCATTCTAAACCTC	pcDNA3.1-IRF3
pcDNA3.1-IRF3-R	CCCAAGCTTGTACAGCTCCATCATCT	
pcDNA3.1-IRF7-F	CGCGGATCCATGGCTCAAAGCCCTCCCAAG	pcDNA3.1-IRF7
pcDNA3.1-IRF7-R	CGGGGTACCATAAAGCTCAGCAGCCAG	
pTurbo-RIP3-F	GGGGTACCGATGGCACTGTCCAGCCAGCT	pTurbo-RIP3-GFP
pTurbo-RIP3-R	CGGGATCCCGGCCAACTCCTCCTGTTTGGT	
qRIP3-F	TCAAGGAAGCAGCCCGTATG	qRT-PCR
qRIP3-R	GAGCCAGTCTGAACACCAACG	
qβ-actin-F	TTATGAAGGCTATGCCCTGCC	qRT-PCR
qβ-actin-R	TGAAGGAGTAGCCACGCTCTGT	

## References

[1] Jorgensen I., Rayamajhi M., Miao E.A. (2017). Programmed cell death as a defence against infection. Nat. Rev. Immunol..

[2] Gan Z., Chen S.N., Huang B., Zou J., Nie P. (2020). Fish type I and type II interferons: Composition, receptor usage, production and function. Rev. Aquac..

[3] Yu Y.Y., Ding L.G., Huang Z.Y., Xu H.Y., Xu Z. (2021). Commensal bacteria-immunity crosstalk shapes mucosal homeostasis in teleost fish. Rev. Aquac..

[4] Brubaker S.W., Bonham K.S., Zanoni I., Kagan J.C. (2015). Innate immune pattern recognition: A cell biological perspective. Annu. Rev. Immunol..

[5] Dolasia K., Bisht M.K., Pradhan G., Udgata A., Mukhopadhyay S. (2018). TLRs/NLRs: Shaping the landscape of host immunity. Int. Rev. Immunol..

[6] He S., Wang X. (2018). RIP kinases as modulators of inflammation and immunity. Nat. Immunol..

[7] Silke J., Rickard J.A., Gerlic M. (2015). The diverse role of RIP kinases in necroptosis and inflammation. Nat. Immunol..

[8] Newton K. (2015). RIPK1 and RIPK3: Critical regulators of inflammation and cell death. Trends Cell Biol..

[9] Sun X., Lee J., Navas T., Baldwin D.T., Stewart T.A., Dixit V.M. (1999). RIP3, a novel apoptosis-inducing kinase. J. Biol. Chem..

[10] Yu P.W., Huang B.C., Shen M., Quast J., Chan E., Xu X., Nolan G.P., Payan D.G., Luo Y. (1999). Identification of RIP3, a RIP-like kinase that activates apoptosis and NFkappaB. Curr. Biol..

[11] Sun L., Wang H., Wang Z., He S., Chen S., Liao D., Wang L., Yan J., Liu W., Lei X. (2012). Mixed lineage kinase domain-like protein mediates necrosis signaling downstream of RIP3 kinase. Cell.

[12] Saleh D., Najjar M., Zelic M., Shah S., Nogusa S., Polykratis A., Paczosa M.K., Gough P.J., Bertin J., Whalen M. (2017). Kinase activities of RIPK1 and RIPK3 can direct IFN-beta synthesis induced by lipopolysaccharide. J. Immunol..

[13] Lawlor K.E., Khan N., Mildenhall A., Gerlic M., Croker B.A., D’Cruz A.A., Hall C., Kaur Spall S., Anderton H., Masters S.L. (2015). RIPK3 promotes cell death and NLRP3 inflammasome activation in the absence of MLKL. Nat. Commun..

[14] Viringipurampeer I.A., Shan X., Gregory-Evans K., Zhang J.P., Mohammadi Z., Gregory-Evans C.Y. (2014). Rip3 knockdown rescues photoreceptor cell death in blind pde6c zebrafish. Cell Death Differ..

[15] Ge Y., Yang H., Zhao L., Luo S., Zhang H., Chen S. (2018). Structural and functional conservation of half-smooth tongue sole *Cynoglossus semilaevis* RIP3 in cell death signalling. Fish Shellfish Immunol..

[16] Dai Y., Cao Y., Chen Z., Huang J., Xiao J., Zou J., Feng H. (2021). RIPK3 collaborates with RIPK1 to inhibit MAVS-mediated signaling during black carp antiviral innate immunity. Fish Shellfish Immunol..

[17] Chen X.H., Lin K.B., Wang X.W. (2003). Outbreaks of an iridovirus disease in maricultured large yellow croaker, *Larimichthys crocea* (Richardson), in China. J. Fish Dis..

[18] Zhang J.T., Zhou S.M., An S.W., Chen L., Wang G.L. (2014). Visceral granulomas in farmed large yellow croaker, *Larimichthys crocea* (Richardson), caused by a bacterial pathogen, *Pseudomonas plecoglossicida*. J. Fish Dis..

[19] Yin F., Gong H., Ke Q., Li A. (2015). Stress, antioxidant defence and mucosal immune responses of the large yellow croaker *Pseudosciaena crocea* challenged with *Cryptocaryon irritans*. Fish Shellfish Immunol..

[20] Zou P.F., Shen J.J., Li Y., Yan Q., Zou Z.H., Zhang Z.P., Wang Y.L. (2019). Molecular cloning and functional characterization of TRIF in large yellow croaker *Larimichthys crocea*. Fish Shellfish Immunol..

[21] Zou P.F., Tang J.C., Li Y., Feng J.J., Zhang Z.P., Wang Y.L. (2021). MAVS splicing variants associated with TRAF3 and TRAF6 in NF-kappaB and IRF3 signaling pathway in large yellow croaker *Larimichthys crocea*. Dev. Comp. Immunol..

[22] Upton J.W., Shubina M., Balachandran S. (2017). RIPK3-driven cell death during virus infections. Immunol. Rev..

[23] Orozco S., Oberst A. (2017). RIPK3 in cell death and inflammation: The good, the bad, and the ugly. Immunol. Rev..

[24] Zou P.F., Shen J.J., Li Y., Zhang Z.P., Wang Y.L. (2020). TRAF3 enhances TRIF-mediated signaling via NF-kappaB and IRF3 activation in large yellow croaker *Larimichthys crocea*. Fish Shellfish Immunol..

[25] Ding Y., Ao J., Chen X. (2017). Comparative study of interleukin-17C (IL-17C) and IL-17D in large yellow croaker *Larimichthys crocea* reveals their similar but differential functional activity. Dev. Comp. Immunol..

[26] Sun M., Mu Y., Ding Y., Ao J., Chen X. (2016). Molecular and functional characterization of Toll-like receptor 21 in large yellow croaker (*Larimichthys crocea*). Fish Shellfish Immunol..

[27] Salinas I., Zhang Y.A., Sunyer J.O. (2011). Mucosal immunoglobulins and B cells of teleost fish. Dev. Comp. Immunol..

[28] Li J., McQuade T., Siemer A.B., Napetschnig J., Moriwaki K., Hsiao Y.S., Damko E., Moquin D., Walz T., McDermott A. (2012). The RIP1/RIP3 necrosome forms a functional amyloid signaling complex required for programmed necrosis. Cell.

[29] Pazdernik N.J., Donner D.B., Goebl M.G., Harrington M.A. (1999). Mouse receptor interacting protein 3 does not contain a caspase-recruiting or a death domain but induces apoptosis and activates NF-kappaB. Mol. Cell. Biol..

[30] Zhang D.W., Lin J.A., Han J.H. (2010). Receptor-interacting protein (RIP) kinase family. Cell. Mol. Immunol..

[31] Ting A.T., Pimentel-Muiños F.X., Seed B. (1996). RIP mediates tumor necrosis factor receptor 1 activation of NF-kappa B but not Fas/APO-1-initiated apoptosis. EMBO J..

[32] He S., Liang Y., Shao F., Wang X. (2011). Toll-like receptors activate programmed necrosis in macrophages through a receptor-interacting kinase-3-mediated pathway. Proc. Natl. Acad. Sci. USA.

[33] Gay N.J., Symmons M.F., Gangloff M., Bryant C.E. (2014). Assembly and localization of Toll-like receptor signalling complexes. Nat. Rev. Immunol..

[34] O’Neill L.A., Bowie A.G. (2007). The family of five: TIR-domain-containing adaptors in Toll-like receptor signalling. Nat. Rev. Immunol..

[35] Ji J., Rao Y., Wan Q., Liao Z., Su J. (2018). Teleost-specific TLR19 localizes to endosome, recognizes dsRNA, recruits TRIF, triggers both IFN and NF-kappaB pathways, and protects cells from grass carp reovirus infection. J. Immunol..

[36] Matsuo A., Oshiumi H., Tsujita T., Mitani H., Kasai H., Yoshimizu M., Matsumoto M., Seya T. (2008). Teleost TLR22 recognizes RNA duplex to induce IFN and protect cells from birnaviruses. J. Immunol..

[37] Chen S.N., Zou P.F., Nie P. (2017). Retinoic acid-inducible gene I (RIG-I)-like receptors (RLRs) in fish: Current knowledge and future perspectives. Immunology.

[38] Mogensen T.H. (2018). IRF and STAT transcription factors—From basic biology to roles in infection, protective immunity, and primary immunodeficiencies. Front. Immunol..

[39] Liu S., Cai X., Wu J., Cong Q., Chen X., Li T., Du F., Ren J., Wu Y.T., Grishin N.V. (2015). Phosphorylation of innate immune adaptor proteins MAVS, STING, and TRIF induces IRF3 activation. Science.

[40] Zou P.F., Chang M.X., Li Y., Xue N.N., Li J.H., Chen S.N., Nie P. (2016). NOD2 in zebrafish functions in antibacterial and also antiviral responses via NF-kappaB, and also MDA5, RIG-I and MAVS. Fish Shellfish Immunol..

[41] Livak K.J., Schmittgen T.D. (2001). Analysis of relative gene expression data using real-time quantitative PCR and the 2(-Delta Delta C(T)) Method. Methods.

[42] Letunic I., Khedkar S., Bork P. (2021). SMART: Recent updates, new developments and status in 2020. Nucleic Acids Res..

[43] Kumar S., Stecher G., Tamura K. (2016). MEGA7: Molecular evolutionary genetics analysis version 7.0 for bigger datasets. Mol. Biol. Evol..

